# Expanding the genetic code: In vivo approaches for incorporating non-proteinogenic monomers

**DOI:** 10.71150/jm.2501005

**Published:** 2025-03-28

**Authors:** Dongheon Lee, Suk Min Yun, Jong-il Choi

**Affiliations:** 1 Department of Biotechnology and Bioengineering, Chonnam National University, Gwangju 61186, Republic of Korea; 2 National Institute of Nakdong Basin Biological Resources, Sangju 37242, Republic of Korea

**Keywords:** genetic code expansion, in vivo, non-proteinogenic monomers

## Abstract

The application of genetic code expansion has enabled the incorporation of non-canonical amino acids (ncAAs) into proteins, introducing novel functional groups and significantly broadening the scope of protein engineering. Over the past decade, this approach has extended beyond ncAAs to include non-proteinogenic monomers (npMs), such as β-amino acids and hydroxy acids. In vivo incorporation of these monomers requires maintaining orthogonality between endogenous and engineered aminoacyl-tRNA synthetase (aaRS)/tRNA pairs while optimizing the use of the translational machinery. This review introduces the fundamental principles of genetic code expansion and highlights the development of orthogonal aaRS/tRNA pairs and ribosomal engineering to incorporate npMs. Despite these advancements, challenges remain in engineering aaRS/tRNA pairs to accommodate npMs, especially monomers that differ significantly from L-α-amino acids due to their incompatibility with existing translational machinery. This review also introduces recent methodologies that allow aaRSs to recognize and aminoacylate npMs without reliance on the ribosomal translation system, thereby unlocking new possibilities for synthesizing biopolymers with chemically diverse monomers.

## Introduction

The canonical genetic code, composed of 64 codons for 20 standard amino acids, restricts the diversity of biopolymers achievable through ribosomal synthesis. To overcome these limitations, scientists have developed strategies to expand the genetic code, enabling the incorporation of non-proteinogenic monomers (npMs) into biopolymers (Costello et al., 2024). These monomers are chemical entities not typically incorporated by the canonical genetic code or used in standard ribosomal synthesis. npMs are promising in biological systems due to their diverse chemical functionalities, which allow for the creation of biopolymers with enhanced or novel properties. Unlike the 20 standard amino acids, npMs offer unique attributes, such as enhanced stability (Humpola et al., 2023; Li et al., 2018), modified electronic properties (Faraldos et al., 2011), studies of post-translational modification (Chen & Tsai, 2022; Gan & Fan, 2024), and additional reactive groups (Saleh et al., 2019; Switzer et al., 2023), expanding the range of chemical modifications possible. These distinct characteristics make npMs valuable in synthetic biology (Krahn et al., 2020; Niu & Guo, 2024), drug development (Ding et al., 2020; Huang & Liu, 2018), biocatalysis (Birch-Price et al., 2024; Lugtenburg et al., 2023) and biomaterials engineering (Chemla et al., 2024; Sisila et al., 2023), allowing researchers to design novel biomolecules with improved stability, pharmacokinetics, or catalytic functions that are difficult to achieve with standard amino acids.

**Fig. 1.**
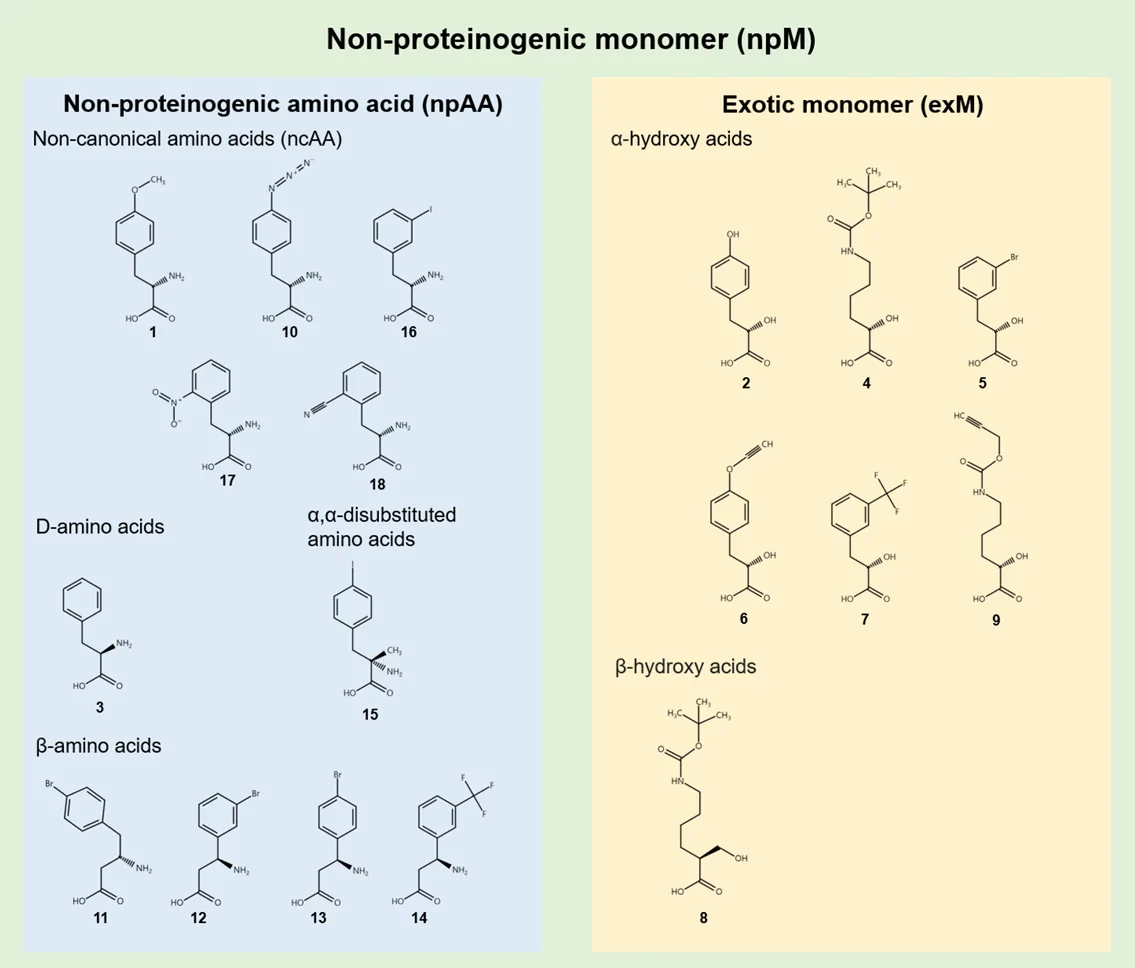
Classification of non-proteinogenic monomers (npMs). Non-proteinogenic monomers (npMs) are categorized into non-proteinogenic amino acids (npAAs) and exotic monomers (exMs). npAAs include non-canonical L-amino acids, D-amino acids, and β-amino acids, while exMs include α- and β-hydroxy acids. The numbers refer to the corresponding chemical structures as indicated in the main text.

npMs can be categorized into non-amino acid exotic monomers (exMs) and non-proteinogenic amino acids (npAAs) (Sigal et al., 2024). npAAs, such as non-canonical L-amino acids, D-amino acids, and β-amino acids, offer new functional groups and expand the stereochemical and structural landscape, while exMs, including α-hydroxy acids and α-thio acids, introduce novel backbones and interactions between monomers that are not achievable using amino acid structures (Fig. 1). This categorization provides a structured approach to capture the diversity of non-canonical monomers, aligning with the overall strategy of expanding the genetic code to incorporate these novel substrates.

Genetic code expansion involves reprogramming translation mechanisms to recognize new codons or synthetic nucleotide pairs, along with engineering translation machinery such as aminoacyl-tRNA synthetases (aaRS), tRNAs, ribosomes, and elongation factors (EFs) to accommodate non-canonical substrates (de la Torre & Chin, 2021; Hammerling et al., 2020; Rodnina, 2018). Expanding the genetic code allows researchers to explore the unique properties of npMs and to create biopolymers with functions and characteristics that exceed those of naturally occurring proteins. Incorporating npMs in vivo presents specific challenges, such as maintaining translation fidelity, ensuring the bioavailability of npMs, and optimizing orthogonal translation systems to function effectively in the cellular environment (Jin et al., 2019). Key components include designing orthogonal aaRS/tRNA pairs that do not interfere with the native machinery of the host and engineering translation components such as ribosomes and elongation factors to handle non-canonical substrates while maintaining cellular fitness (Arranz-Gibert et al., 2019). Addressing these challenges requires both rational design and directed evolution to ensure the expanded genetic code operates efficiently in living cells.

Advances in genetic code expansion have led to several key developments, including orthogonal translation systems, codon expansion techniques, and the development of genomically recoded organisms (GROs) (Chemla et al., 2018; Guo & Niu, 2022; Shandell et al., 2021). These advancements have enabled the successful incorporation of a diverse range of npMs into in vivo systems, which have broadened the range of npMs that can be incorporated through recent improvements in efficiency and specificity.

This review provides an overview of the strategies and challenges associated with incorporating npMs through genetic code expansion, with a focus on in vivo approaches. It explores the use of diverse orthogonal aaRS/tRNA pairs for incorporating npMs, while excluding non-canonical L-amino acids. Additionally, this review introduces methods for engineering orthogonal aaRS capable of recognizing novel npMs independently of the ribosomal translation mechanism.

## Overview of Genetic Code Expansion

Expanding the genetic code to incorporate npMs involves modifying and optimizing the translation mechanism and its core components, as well as developing orthogonal translation systems (OTS). The translation mechanism focuses on how engineered components, such as aaRSs, tRNAs, ribosomes, and elongation factors, are adapted to incorporate npMs (Kim et al., 2022). OTS are designed to function independently of the natural translation machinery of the host, providing a robust framework for incorporating npMs with high specificity and minimal competition with standard amino acids (Fu et al., 2022).

**Fig. 2.**
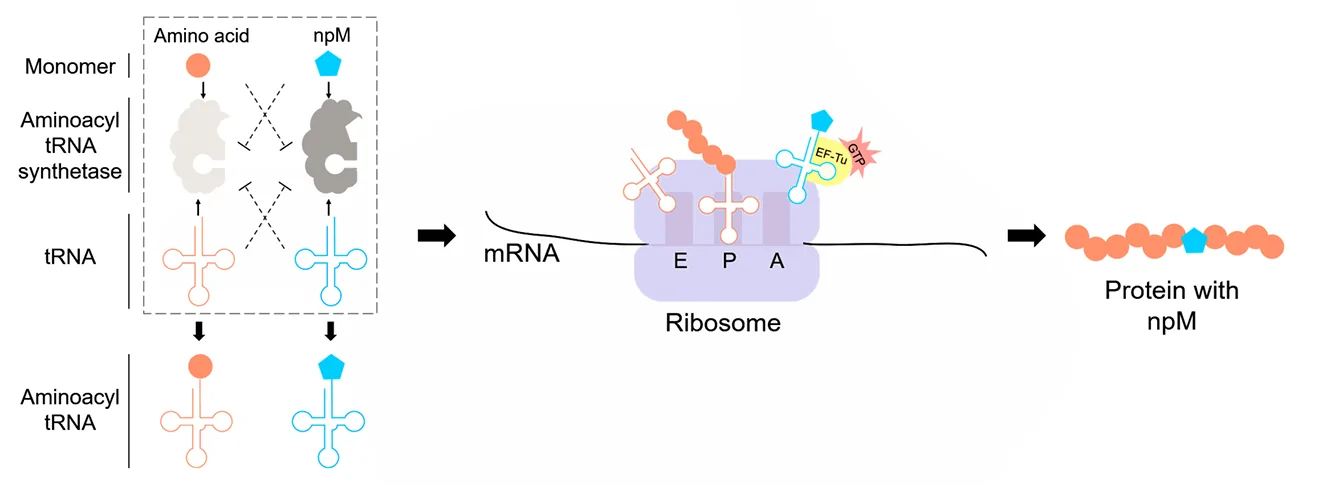
Schematic representation of the orthogonal translation system for the in vivo incorporation of non-proteinogenic monomers (npMs). Orthogonal aminoacyl-tRNA synthetases (aaRS) and their corresponding tRNAs are engineered to aminoacylate non-proteinogenic monomers (npMs) while avoiding interference from endogenous aaRS/tRNA pairs and canonical amino acids. The aminoacylated tRNAs carrying npMs are delivered to the ribosome, where they occupy the A-site through interactions with elongation factor Tu (EF-Tu) and GTP. The translation process incorporates the npMs into the growing peptide chain, resulting in novel biopolymers with expanded chemical diversity.

The successful incorporation of npMs relies on engineering and optimizing core components involved in the translation process. The translation mechanism begins with aaRS, which charges the tRNA with npMs (Fig. 2). Once charged, the modified tRNA recognizes specific codons, delivering the monomer to the ribosome. The ribosome then facilitates translation by incorporating the monomer into the growing polypeptide chain. Throughout this process, elongation factors, such as EF-Tu, ensure that the npM-charged tRNA is positioned correctly and that translation proceeds efficiently (Xu et al., 2021). These components work together to expand the genetic code and incorporate npMs into proteins.

### Orthogonal Translation Systems (OTS)

Orthogonal translation systems are engineered to function independently of the natural translation machinery of the host. They use orthogonal aaRS and tRNA pairs that specifically recognize and incorporate npMs without interfering with endogenous translational components. OTS are an effective approach for incorporating npMs, ensuring high specificity and reducing competition with natural amino acids.

Various aaRS/tRNA pairs have been developed to incorporate npMs into proteins. To avoid interference with endogenous aaRS or tRNA, orthogonal aaRS/tRNA pairs from various foreign species have been introduced and manipulated. A representative strategy involves utilizing variations in recognition mechanisms among tRNA homologs from different domains to create orthogonal aaRS/tRNA pairs (Melnikov & Söll, 2019). This strategy has been successfully implemented by transplanting aaRS/tRNA pairs from archaea into different domains, including bacteria, eukaryotes, and mammalian cells. Notable examples include the use of *Methanococcus jannaschii* TyrRS/tRNA^Tyr^ and *Methanosarcina* species PylRS/tRNA^Pyl^ pairs. For instance, *M. jannaschii* TyrRS/tRNA^Tyr^ (Amiram et al., 2015; Wang et al., 2001), *Methanosarcina barkeri* PylRS/tRNA^Pyl^ (Blight et al., 2004; Neumann et al., 2008), *Methanosarcina mazei* PylRS/tRNA^Pyl^ (Neumann et al., 2008; Yanagisawa et al., 2008b), and *Methanomethylophilus alvus* PylRS/tRNA^Pyl^ (Beránek et al., 2019; Willis & Chin, 2018) are widely used. Additionally, orthogonal aaRS/tRNA pairs such as *Saccharomyces cerevisiae* PheRS/tRNA^Phe^ (Furter, 1998; Kwon et al., 2006), TrpRS/tRNA*Trp* (Chatterjee et al., 2013), and *Pyrococcus horikoshii* LysRS/tRNA^Lys^ (Anderson et al., 2004) have been studied. These examples demonstrate the growing repertoire of tools available for expanding the genetic code, which facilitate the incorporation of increasingly complex npMs.

### Stop codon, quadruplet codon, and sense codon suppression in genetic code expansion

Expanding the genetic code to incorporate npMs involves reassigning existing codons or introducing new coding strategies to overcome the limitations of the standard genetic code. Two widely used strategies for this purpose are stop codon suppression and the introduction of quadruplet codons. Stop codon suppression reassigns one of the three natural stop codons—amber (UAG), ochre (UAA), and opal (UGA)—for the purpose of encoding npMs. Among these, the amber codon is most commonly used, particularly in *Escherichia coli*, as it is the least frequently utilized stop codon in this model organism (Nakamura et al., 2000).

An early study investigated the *M. jannaschii* TyrRS/tRNA^Tyr^ pair, in which the anticodon of tRNA^Tyr^ (GUA), originally recognizing a tyrosine codon, was replaced with CUA to recognize the amber codon UAG (Wang & Schultz, 2001). This modification enabled the incorporation of O-methyl-L-tyrosine 1 at amber codon sites in *E. coli* proteins (Wang et al., 2001). Since then, numerous studies have refined amber suppression by developing enhanced orthogonal aaRS/tRNA pairs and improving translation efficiency (Kato, 2019). Another example utilizes the PylRS/tRNA^Pyl^ pair, which naturally recognizes the UAG codon (Srinivasan et al., 2002). The PylRS/tRNA^Pyl^ pair achieves orthogonality through unique structural features, such as a compacted binding site in the synthetase and distinctive structural elements in tRNA^Pyl^, including a shortened variable loop and elongated anticodon stem (Nozawa et al., 2009; Srinivasan et al., 2002). These adaptations prevent recognition by the endogenous aminoacyl-tRNA synthetases and tRNAs, to ensure precise interaction exclusively with the amber codon.

Despite its success, stop codon suppression is inherently limited by the small number of available nonsense codons. To address this limitation, quadruplet codons have been explored as a strategy to increase the coding capacity and to enable the simultaneous incorporation of multiple npMs into a single protein. Early research in *E. coli* demonstrated the potential of quadruplet codons for genetic code expansion. For example, one study showed that mutant tRNA^Leu^ with engineered anticodon loops could decode specific quadruplet codons such as UAGA, enabling the incorporation of additional amino acids into proteins (Anderson et al., 2004). Another study achieved ncAA incorporation in response to quadruplet codons by engineering tRNA^Lys^ anticodon loops to target specific frameshift codons, such as AGGA, enabling dual ncAA incorporation into proteins (Moore et al., 2000). A recent study demonstrated the synthesis of protein containing four distinct ncAAs at quadruplet codon sites using four mutually orthogonal aaRS/tRNA pairs. This achievement underscores the growing potential of quadruplet codons for expanding the repertoire of encoded monomers (Dunkelmann et al., 2021).

In addition to nonsense and quadruplet codon strategies, other strategies have been developed to explore the reassignment of sense codons to ncAAs. Researchers modified the anticodon loop of *M. jannaschii* tRNA^Tyr^ and its cognate aaRS, reassigning the AGG codon, which encodes arginine, to tyrosine. This approach enhanced the efficiency of tyrosine incorporation and showed similar improvements for ncAA incorporation, highlighting the versatility of sense codon reassignment as a complementary strategy for genetic code expansion (Biddle et al., 2022). Another study focused on incorporating monomers into serine codons. By utilizing engineered *M. mazei* PylRS/tRNA^Pyl^ pairs in combination with genomically recoded organism (GRO), ncAAs were successfully incorporated into the serine codons TCG and TCA (Robertson et al., 2021). GRO provides a versatile platform for enhancing the efficiency of amber codon incorporation and facilitating sense codon reassignment, which will be discussed further in the next chapter. This process involves removing the tRNAs that decode these codons and leveraging orthogonal translational machinery to achieve high specificity in ncAA incorporation.

### Genomically recoded organisms

Recent advancements in genetic code expansion have enabled the study of reassigning the genetic code within the entire genome. These efforts have resulted in the creation of genomically recoded organisms (GROs), which are designed and synthesized to use an alternate genetic code for enhanced flexibility and novel functionalities in protein synthesis. GROs are engineered by systematically replacing codons across the genome and manipulating translational components, enabling precise control over genetic coding and allowing the incorporation of npMs with minimal competition from endogenous machinery.

A notable example is the C321 strain, derived from *Escherichia coli* MG1655 through conjugative assembly genome engineering (CAGE) (Isaacs et al., 2011). This method enables the systematic transfer of synthesized chromosomal regions from a donor to a recipient, resulting in a fully recoded genome (Lajoie et al., 2013). In the C321 strain, all 321 UAG stop codons were replaced with UAA codons. To further enhance amber suppression, the *prfA* gene, which encodes release factor 1 responsible for UAG recognition during translational termination, was deleted to produce the C321.ΔA strain. However, this process introduced 355 off-target mutations, resulting in a 60% increase in doubling time compared to the original MG1655 strain. To address this issue, researchers used multiplex automated genome engineering (MAGE), a technique for simultaneous multiple genetic modifications, combined with whole-genome sequencing to identify and correct fitness-related mutations (Wang et al., 2009). This approach successfully modified six critical mutations, leading to the development of the C321.ΔA opt strain, which recovered 59% of the fitness observed in the C321.ΔA strain (Kuznetsov et al., 2017).

As another example, GRO research has expanded to include the systematic reassignment of sense codons. One notable example is the *E. coli* MDS42 strain, characterized by a simplified genome with the removal of non-essential genes. In this strain, the serine codons TCG and TCA were systematically replaced with their synonymous counterparts, AGC and AGT, respectively, in the genome. Subsequently, the *prfA* gene, which is responsible for terminating translation at the amber codon, and the *serU* and *serT* tRNA genes, which decode TCG and TCA, were deleted. This led to the creation of the Syn61 strain, a streamlined organism that uses only 61 codons, excluding the amber codon and two serine codons, thereby expanding the potential for blank codon usage and novel synthetic biology applications (Fredens et al., 2019).

These advancements in GRO development demonstrate the potential of genome-wide codon reassignment in genetic code expansion. By creating organisms with optimized genetic codes, researchers can introduce novel functionalities, improve translation fidelity, and provide unique platforms for incorporating non-proteinogenic monomers into proteins.

## Engineering Translation Components for Non-proteinogenic Monomer Incorporation

The incorporation of npMs into proteins is important for expanding the chemical and functional diversity of biomolecules. To achieve this, engineering of translational components is crucial for maintaining specificity, efficiency, and fidelity. Advancements in aaRSs, tRNAs, and ribosomes have expanded the repertoire of npMs that can be incorporated. In this section, we focus on the in vivo approaches for incorporating npMs, excluding non-canonical L-α-amino acids, which are extensively covered in reviews (Ishida et al., 2024; Rezhdo et al., 2019; Tang et al., 2022).

### Engineered aminoacyl-tRNA synthetase/tRNA pair

The genetic incorporation of npMs in vivo often relies on the engineering aaRS and their cognate tRNAs to achieve substrate specificity and translational fidelity. For instance, the α-hydroxy acid, p-hydroxy-L-phenyllactic acid 2, was incorporated into proteins in *E. coli* using an engineered *M. jannaschii* tyrosyl-tRNA synthetase (Guo et al., 2008). To achieve this, five residues within the active site were randomized. Positive selection was then implemented to identify mutants capable of activating the desired substrate and negative selection to remove those that recognize endogenous amino acids (Wang et al., 2006). After screening, a synthetase variant capable of incorporating the hydroxy acid at amber codon sites in the myoglobin protein was identified.

Similarly, a study utilizing a polysubstrate-specific aminoacyl-tRNA synthetase, pCNFRS, developed from the same *M. jannaschii* derived TyrRS/tRNA^Tyr^ pair, successfully demonstrated the site-specific incorporation of eight D-AAs, including D-Phe, D-Asn, D-Ile, D-Met, D-Arg, D-Ala, D-Pro, and D-Val, into GFPuv at residue 18, which had been mutated to an amber stop codon (Liu et al., 2012; Young et al., 2011). Additionally, D-Phe 3 was specifically introduced at the fluorophore-forming Tyr66 residue of GFPuv, resulting in a mutant protein with shifted emission and excitation wavelengths and enhanced thermal stability compared to its L-isomer counterpart.

The PylRS from *M. mazei* has also shown remarkable versatility in incorporating npMs by leveraging its loose recognition of the α-amino group (Kobayashi et al., 2009; Yanagisawa et al., 2008a). PylRS successfully acylated α-hydroxy acids such as Boc-LysOH 4 onto tRNA^Pyl^, demonstrating high efficiency, with yields surpassing those of Boc-lysine. Sterically hindered substrates like NMe-Boc-lysine and D-Boc-lysine showed reduced activity, likely due to substrate-binding pocket clashes. The successful incorporation of Boc-LysOH 4 into proteins at amber codon sites in *E. coli* further established the utility of this system for site-specific introduction of ester bonds.

Additionally, PylRS/tRNA^Pyl^ systems have been employed to streamline therapeutic peptide production through the incorporation of hydroxy acids. For instance, Boc-LysOH 4, HO-Phe(3-Br)-OH 5, and HO-Tyr(propargyl)-OH 6 were site-specifically introduced into lanthipeptides like lacticin 481 and nukacin ISK-1 in *E. coli* using an amber codon suppression system (Bindman et al., 2015). This enabled the formation of ester bonds, facilitating the efficient removal of leader peptides under alkaline conditions without proteolytic enzymes, which are often limited by sequence specificity.

Similarly, recent advancements have enabled the in vivo incorporation of α-hydroxy acids into proteins using wild-type and engineered pyrrolysyl-tRNA synthetase systems from *M. alvus* (MaPylRS), achieving site-specific incorporation of monomers such as Boc-LysOH 4 and α-OH-m-CF3-Phe 7, respectively, into superfolder GFP at amber codon sites in *E. coli* (Fricke et al., 2023; Wang et al., 2011, 2012). Additionally, engineered derivatives were also shown to selectively acylate npMs in vitro, including α-hydroxy acids, α-thio acids, N-formyl-L-α-amino acids, and α-carboxy acids. Structural analysis of the engineered derivative MaFRSA bound to the prochiral m-CF3-2-benzylmalonate, an α-carboxy acid, revealed that PylRS adapts to the expanded size of the α-substituent by forming new stabilizing hydrogen bonds with the α-carboxy group. It also employs distinct binding modes for the pro-S and pro-R configurations of the substrate (Fricke et al., 2023). Based on these findings, subsequent studies demonstrated the in vivo incorporation of multiple (S)-β^2^-hydroxy acids into proteins using the orthogonal MaPylRS/MatRNA^Pyl^ system (Hamlish et al., 2024). The MaPylRS enzyme demonstrated a preference for (S)-β^2^-hydroxy acids as substrates in cellulo, a finding attributed to their absolute configuration aligning with that of D-α-amino acids. In vivo synthesis of protein containing two (S)-β^2^-OH 8, a β^2^-hydroxy acid whose backbone was extended to a β^2^ configuration from Boc-LysOH 4, was confirmed. This work represents the first successful in vivo synthesis of proteins containing multiple β^2^-hydroxy acids, and thus expanded the chemical diversity of engineered proteins.

Additionally, two distinct non-proteinogenic monomers, non-canonical amino acids and hydroxy acids, were incorporated into macrocyclic peptides in *E. coli* (Spinck et al., 2023). These macrocycles were synthesized in a Syn61-derived strain (Robertson et al., 2021), a GRO with reduced codon redundancy. This strain enables the suppression of the UAG stop codon as well as the UCG and UCA sense codons, facilitating the efficient incorporation of multiple monomers. For example, this was achieved using mutually orthogonal pairs, such as the *M. mazei* PylRS/tRNA^Pyl^ for the UCG codon to incorporate AlkynK-OH 9 and the *Archaeoglobus fulgidus* TyrRS/tRNA^Tyr^ for the UAG codon to incorporate para-azido-L-phenylalanine 10. The resulting macrocycles featured ester bonds and novel side chains, demonstrating the potential for incorporating multiple chemically diverse npMs and expanding the range of non-canonical polymers.

### Ribosome engineering

A strategy for incorporating npMs into proteins involves engineering ribosomes to accommodate non-canonical backbones. This is often achieved by increasing the flexibility of the peptidyltransferase center (PTC) within the ribosome, enabling the accommodation of npMs. Antibiotics such as erythromycin and puromycin, which mimic substrates and are recognized at the PTC, can be employed to facilitate selection for desired traits during engineering (Hecht, 2022).

Building on the 040329 ribosome, which was optimized for β-amino acid incorporation in vitro through specific mutations in the 23S rRNA (Dedkova et al., 2012), a more advanced ribosome named P7A7 was developed (Melo Czekster et al., 2016). Targeted mutations in the A-site-adjacent region of the 23S rRNA (positions 2502–2507) enhanced its efficiency in incorporating β^3^-(p-Br)Phe 11. When paired with the *E. coli* PheRS/tRNA^Phe^ system and the wild-type elongation factor Tu (EF-Tu), the P7A7 ribosome demonstrated a threefold improvement in β^3^-Phe incorporation efficiency compared to the 040329 ribosome, while simultaneously achieving a more favorable incorporation ratio of β^3^-(p-Br)Phe to α-Phe. However, further analysis revealed that the P7A7 ribosome faced structural and assembly challenges, with disordered regions in the PTC and adjacent helices leading to inefficient 70S ribosome formation (Ward et al., 2019). These structural instabilities also led to subunit association deficiencies, requiring higher magnesium concentrations to stabilize the ribosome complex. These findings emphasize the importance of balancing functionality and stability in the design of ribosomes for non-canonical translation systems.

## Emerging Strategies for Developing Orthogonal Translation Systems to Incorporate npMs

The expansion of the genetic code to incorporate npMs in vivo has been driven by OTS, which use engineered aaRS/tRNA pairs and modified ribosomes. However, traditional methods for developing OTS to incorporate exotic monomers or non-proteinogenic amino acids such as positive and negative selection methods (Guo et al., 2008; Wang & Schultz, 2001) often rely on translation-dependent readouts. This dependence restricts the range of compatible monomers and complicates screening procedures, as the strong coupling between ribosomal translation efficiency and the specificity of the synthetase for monomers can create bottlenecks, particularly with structurally diverse, less compatible substrates exhibiting low fidelity. To address these challenges, recent studies have introduced more flexible and efficient strategies for OTS development. These approaches aim to expand the chemical diversity accessible through in vivo genetic code expansion while reducing the constraints imposed by traditional, translation-dependent methodologies.

**Fig. 3.**
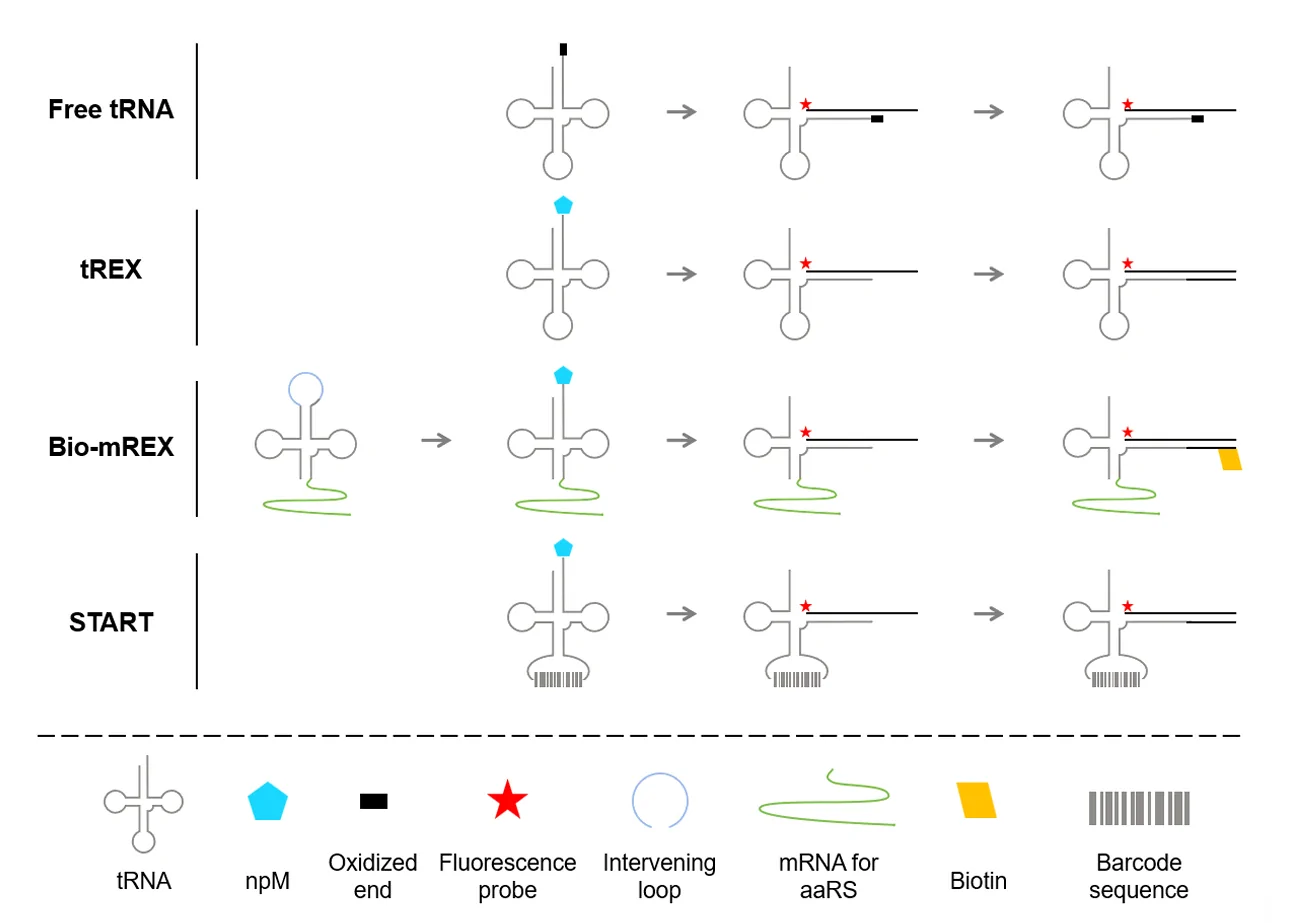
Overview of translation-independent tRNA acylation and screening methodologies. To engineer aminoacyl-tRNA synthetases (aaRSs) or screen orthogonal aaRS/tRNA pairs, translation-independent methodologies have been utilized. These methods exploit the chemical distinctions between aminoacylated and non-aminoacylated tRNAs to enable precise selection and analysis. Non-aminoacylated tRNAs are selectively oxidized at their 3′ end, rendering them incapable of extension, while aminoacylated tRNAs remain unmodified, allowing subsequent extension. tREX facilitates screening by differentiating aminoacylated tRNAs through either by size separation via gel electrophoresis or by using biotinylated oligonucleotides. Building upon tREX, the bio-mREX approach improves screening efficiency by linking mRNAs encoding aaRS variants to their corresponding tRNAs. This technique employs split tRNAs with an intervening loop to assign specific mRNAs to each tRNA, and utilizes biotinylated extensions on aminoacylated tRNAs, which can then be captured using streptavidin beads. The START methodology employs sequence-barcoded tRNAs by assigning unique barcodes to the anticodon region, as PylRS tolerates additional nucleotides. This approach utilizes next-generation sequencing (NGS) to directly connect the genotype of aaRS mutants to their acylation activity, enabling high-throughput analysis and facilitating directed evolution.

The tRNA Extension (tREX) methodology introduces an efficient, scalable approach to identify and evaluate orthogonal aaRS/tRNA pairs for the genetic incorporation of npMs (Cervettini et al., 2020). At its core, tREX leverages a two-step biochemical strategy to determine the aminoacylation status of candidate tRNAs (Fig. 3). First, tRNA is subjected to selective oxidation of its 3′ terminal hydroxyl group. This treatment prevents extension by polymerase unless the tRNA is aminoacylated, preserving the attachment of the amino acid. Second, a Cy5-labeled DNA probe complementary to the tRNA acceptor stem is hybridized, and the resulting hybrid is extended using a 3′-to-5′ exonuclease-deficient Klenow fragment of DNA polymerase I. Only aminoacylated tRNAs resist oxidation and undergo extension, enabling precise differentiation between charged and uncharged tRNAs via native PAGE. Using this method, tREX successfully identified eight mutually orthogonal aaRS-tRNA pairs that function independently of endogenous *E. coli* pairs.

Expanding on the earlier fluoro-tREX method, the bio-mREX approach was specifically developed to improve the efficiency of synthetase mutant screening, allowing for the identification of variants capable of efficiently acylating tRNAs (Dunkelmann et al., 2024). This progression included the introduction of split tRNA (stRNA) systems, initially employing trans configurations where tRNA halves divided at the anticodon were expressed separately and assembled in vivo to enable modular testing of tRNA functionality and flexibility in structural design (Fig. 3). This process was further refined in the cis stRNA approach, by circularly permuting the tRNA gene and using intervening sequences processed by endogenous RNases, ensuring efficient assembly and tRNA reconstitution. Building on this foundation, stmRNA were developed to integrate one half of the cis stRNA with the synthetase mRNA, creating a system that co-expressed both components and enabled aminoacylation via the synthetase encoded on its linked mRNA transcript. Bio-mREX utilized biotin-labeled probes rather than fluorescent probes, and streptavidin beads to selectively capture acylated tRNAs, which were then converted into cDNA for targeted synthetase mutant screening. This process, combined with next-generation sequencing (NGS), allowed for high-throughput screening and detailed analysis of synthetase-tRNA pairs, facilitating the identification of synthetases compatible with structurally diverse non-proteinogenic monomers. Furthermore, the tRNA display technology enabled by bio-mREX expanded the scope of genetic code incorporation to include not only L-α-amino acids but also a wide range of structurally diverse non-proteinogenic monomers. These included β-amino acids such as (S)-3-amino-3-(3-bromophenyl)propanoic acid 12, (S)-3-Amino-3-(4-bromophenyl)propanoic acid 13, and (S)-3-Amino-3-[3-(trifluoromethyl)phenyl]propanoic acid 14, as well as α,α-disubstituted amino acids like (S)-alpha-Methyl-4-Iodophenylalanine 15. This achievement demonstrates the versatility of the system in accommodating chemically and structurally unique substrates, opening new opportunities for developing aaRS variants for novel monomers.

As a similar approach, the START (Sequence-based Translation-independent Acylation of RNA by aaRSs with barcoding for Translation) methodology also emphasizes translation-independent strategies to engineer aaRSs by directly linking tRNA acylation to aaRS activity through a sequence-based barcoding system (Soni et al., 2024). In this strategy, candidate aaRS mutants acylate barcode-tagged tRNAs, with their activity subsequently analyzed through high-throughput sequencing. A key feature of the START methodology is its reliance on barcode-tagged tRNAs, each uniquely linked to a specific aaRS mutant (Fig. 3). By utilizing the anticodon loop of tRNA as a permissive site for sequence barcoding, which is possible because PylRS does not interact with the anticodon region and tolerates expanded tRNA^pyl^ anticodons, the system maintains efficient tRNA identification (Ambrogelly et al., 2007; Nozawa et al., 2009). This design allows for the creation of a highly diverse barcode library, with 10 randomized nucleotides generating 1 × 10^6^ unique sequences. To further facilitate the screening process, polymerase-mediated extension of the 3′-terminus is employed using a DNA template hybridized onto the tRNA sequence. This step allows selective tagging of acylated tRNAs by hybridizing a DNA template to the intact 3′-terminus, enabling their subsequent enrichment and identification through reverse transcription (RT) PCR. By minimizing interference from endogenous translational machinery, this translation-independent approach is effective for screening synthetase mutants with non-standard or structurally complex substrates. Each barcode establishes a direct link between genotype and phenotype, allowing identification of active variants through NGS. Using the START strategy, researchers successfully identified aminoacyl-tRNA synthetase mutants capable of incorporating ncAAs such as m-iodo-L-phenylalanine 16, o-nitro-L-phenylalanine 17 and o-cyano-L-phenylalanine 18. Additionally, the study confirmed that the START system is compatible with Boc-LysOH 4, demonstrating its scalability for incorporating diverse npMs.

A key distinction between these approaches is that tREX, bio-mREX, and START differ in their strategies for identifying and optimizing orthogonal aaRS/tRNA pairs for genetic code expansion. tREX utilizes tRNA oxidation and fluorescence labeling to distinguish aminoacylated tRNAs from non-aminoacylated ones, enabling the rapid screening of orthogonal pairs without direct dependence on translation. Building upon tREX, bio-mREX enhances this approach by integrating mRNA-tRNA pairing through split tRNA systems, allowing for high-throughput selection using biotin-based capture and NGS analysis. This advancement enables the efficient screening of synthetase variants with improved substrate specificity. Meanwhile, START introduces a unique barcode-based system, where sequence tags in the anticodon loop of tRNAs are used to track acylation events via NGS. This method ensures a direct genotype-phenotype link, allowing for large-scale mutational analysis and precise optimization of aaRS variants. While all three approaches facilitate the identification of synthetase mutants capable of incorporating npMs, bio-mREX and START offer higher throughput and scalability compared to tREX, making them suitable for the rapid evolution of translation systems, particularly aaRS, to accommodate chemically diverse substrates.

These emerging methodologies, including tREX, bio-mREX, and START, collectively illustrate the transformative potential of translation-independent and high-throughput strategies for engineering orthogonal translation systems. By addressing the limitations of traditional, translation-dependent approaches, these methods are expected to enable the incorporation of structurally diverse and chemically unique non-proteinogenic monomers, significantly expanding the chemical diversity accessible through genetic code expansion.

## Conclusions and Future Perspectives

This review highlights the remarkable advancements in genetic code expansion, particularly focusing on the in vivo incorporation of non-proteinogenic monomers (npMs) into proteins. By leveraging orthogonal translation systems (OTS), including engineered aminoacyl-tRNA synthetase (aaRS)/tRNA pairs, codon reassignment, and genomically recoded organisms, researchers have significantly advanced the field of genetic code expansion. In particular, the development of engineered translational components, such as aaRS/tRNA pairs and ribosomes, has facilitated the incorporation of chemically and functionally diverse npMs, including α-hydroxy acids, β-amino acids, and other monomers, in vivo through ribosomal translation. Furthermore, recent methodologies such as tREX, bio-mREX, and START have addressed key challenges in translational dependency of aaRS/tRNA engineering on ribosome-mediated translation by introducing translation-independent approaches.

Despite these significant advancements, the practical applications of npMs, excluding non-canonical L-α-amino acids, have been largely studied in vitro, employing techniques such as solid-phase peptide synthesis (SPPS) or flexible in vitro translation (FIT) systems (Goto et al., 2011; Palomo, 2014), and, to the best of our knowledge, have remained limited in vivo. Although in vitro integration has demonstrated promising potential (Table 1), realizing this potential in vivo remains challenging, particularly with regard to achieving efficient incorporation into proteins in living systems, necessitating further expansion of the substrate scope. To address this, translation-independent screening methods—tREX, bio-mREX, and START—are anticipated to accelerate the discovery of novel aaRS/tRNA pairs and broaden the substrate scope. In parallel, computational approaches, such as predictive modeling of substrate binding affinities (Ren et al., 2015) and rational design of aaRS (Baumann et al., 2019), are expected to facilitate the development of translation components, enabling more precise and scalable in vivo incorporation of npMs.

One potential future direction involves developing microbial platforms specifically optimized for non-natural polymer production. By engineering microbial cell factories to metabolically produce desired npMs alongside optimized genetic codes and translational machinery, the biosynthesis of non-natural polymers could be significantly improved. Additionally, novel enzymes capable of catalyzing unprecedented chemical reactions might be developed. Integrating npMs with atoms such as sulfur, chlorine, or phosphorus—which possess unique polarities and sizes—could enable the design of artificial active sites and create innovative catalytic reactions. Lastly, predictive models capable of accommodating complex polymers with novel side chains and backbones are required to fully design npM-incorporated biopolymers, as current protein structure prediction models primarily rely on databases of known protein structures (Niazi et al., 2024) and are limited when applied to non-canonical proteins.

In conclusion, as the genetic code expansion continues to evolve, it will likely serve as an innovative technology for overcoming the limitations of traditional protein engineering, synthesizing novel biopolymers, and opening new frontiers in biotechnology.

## Figures and Tables

**Table 1.** Engineered peptides for practical applications through in vitro integration of non-proteinogenic monomers

Application	Engineered peptide	Introduced npMs	Additional features	Method	Ref
Biomaterial	D-peptide hydrogelator	D-Phe, D-Lys, D-Tyr phosphate	Phosphatase substrate compatibility, resistant to proteolytic degradation	SPPS	Li et al. (2013)
	Collagen mimetic peptide	Aza-glycine	Triple-helix thermal stability	SPPS	Zhang et al. (2015)
	Peptide linker for hydrogels	D-Val, D-Pro, D-Met, D-Ser, D-Arg	Resistance to enzymatic degradation, increased cytotoxicity	SPPS	Bomb et al. (2023)
Therapeutic	Agonist of GLP-1 receptor	cyclic β-amino acids	Native-like potency, proteolysis resistance	SPPS	Johnson et al. (2014)
	Aurein 1.2 peptide analog	β-Leu, β-Lys, β-Ile, β-Ala, β-Ser, β-Phe, cyclic β-amino acids	Increased helical stability, improved selectivity, reduced hemolysis	SPPS	Lee et al. (2017)
	Ubiquitin chain binding peptide	D-Ala, D-Phe, N-methyl-Gly, N-methyl-Ala	High affinity, protease resistance, membrane permeability	FIT	Rogers et al. (2021)
	Ganglioside GM1-binding peptide tag	β-Trp, β-Tyr, β-Lys	Binding affinity, protease resistance	SPPS	Hetényi et al. (2022)
	Inhibitor of SARS-CoV-2 main protease	cyclic γ-amino acids, cyclic β-amino acids	High affinity, proteolytic stability, prolonged serum stability	FIT	Miura et al. (2023, 2024b)
	Inhibitor of interferon gamma receptor 1	cyclic β-amino acids, cyclic γ-amino acids	Enhanced binding affinity, inhibitory activity, prolonged serum stability	FIT	Miura et al. (2024a)
